# Male infertility is associated with differential DNA methylation signatures of the imprinted gene GNAS and the non-imprinted gene CEP41

**DOI:** 10.1007/s10815-024-03202-w

**Published:** 2024-07-17

**Authors:** Suheyla Esra Ozkocer, Ismail Guler, Asiye Ugras Dikmen, Nuray Bozkurt, Nuray Varol, Ece Konac

**Affiliations:** 1 Department of Medical Biology and Genetics, Institute of Health Sciences, Gazi University, Kavaklıdere Çankaya, 06540 Ankara, Turkey; 2 Department of Histology and Embryology, Faculty of Medicine, Gazi University, Besevler, 06500 Ankara, Turkey; 3 Department of Obstetrics and Gynecology, Faculty of Medicine, Gazi University, Besevler, 06500 Ankara, Turkey; 4 Department of Public Health, Faculty of Medicine, Gazi University, Besevler, 06500 Ankara Turkey; 5 Department of Medical Biology, Faculty of Medicine, Gazi University, Besevler, 06500 Ankara, Turkey

**Keywords:** *CEP41*, DNA methylation, Epigenetic, Expression levels, *GNASAS*, Male infertility

## Abstract

**Purpose:**

To investigate whether the DNA methylation profiles of *GNAS(20q13.32)*, *MEST(7q32.2)*, *MESTIT1(7q32.2), IGF2(11p15.5)*, *H19 (7q32.2)*, and *CEP41(7q32.2)* genes are related to the transcriptomic and epigenomic etiology of male infertility.

**Methods:**

The DNA methylation levels of spermatozoa were obtained from fertile (*n* = 30), oligozoospermic (*n* = 30), and men with normal sperm count (*n* = 30). The methylation status of each CpG site was categorized as hypermethylated or hypomethylated. Expression levels of target gene transcripts were determined using real-time PCR.

**Results:**

The oligozoospermia showed a higher frequency of hypermethylation at *GNASAS* 1st, 3rd, and 5th CpG dinucleotides (66.7%, 73.3%, 73.3%) compared to the fertile group (33.3%, 33.3%, 40%, respectively). The normal sperm count exhibited a higher frequency of hypermethylation at the 3rd CpG of *CEP41* (46.7%) than the fertile group (16.7%). Normal sperm count was predicted by *CEP41* hypermethylation (OR = 1.750, 95%CI 1.038–2.950) and hypermethylation of both *CEP41* and *GNASAS* (OR = 2.389, 95%CI 1.137–5.021). Oligozoospermia was predicted solely by *GNASAS* hypermethylation (OR = 2.460, 95%CI 1.315–4.603). In sperms with decreased *IGF2* expression in the fertile group, we observed hypomethylation in the 2nd CpG of *IGF2* antisense *(IFG2AS)*, and hypermethylation in the 1st, 2nd, and 4th CpGs of *H19*. No significant relationship was found between *IGF2* expression and methylation status of *IGF2AS* and *H19* in infertile groups.

**Conclusion:**

The disappearance of the relationship between *IGF2* expression and *IGF2AS* and *H19* methylations in the infertile group provides new information regarding the disruption of epigenetic programming during spermatogenesis. A better understanding of sperm *GNASAS* and *CEP41* hypermethylation could advance innovative diagnostic markers for male infertility.

**Supplementary Information:**

The online version contains supplementary material available at 10.1007/s10815-024-03202-w.

## Introduction

Infertility is the problem of a couple who cannot achieve pregnancy after unprotected sexual intercourse [1]. Male infertility prevalence can differ over time and contributes to almost half of the cases [2]. Semen analysis is the initial test to evaluate male infertility, with the World Health Organization (WHO) establishing the 5th percentile values of the population as limits for parameters like sperm count, motility, and morphology. Poor semen parameters are categorized based on conditions such as oligozoospermia (low sperm count), teratozoospermia (abnormal morphology), and asthenozoospermia (low sperm motility). While poor semen parameters indicate reduced chances of spontaneous pregnancy, normal values do not exclude infertility. Oligozoospermia indicates sperm concentrations lower than the WHO’s criteria; normozoospermia means the values are within normal limits [3]. Since over one-third of male infertility cases are idiopathic [2], genetic and epigenetic factors have the potential to identify underlying causes [4, 5].

Assisted reproductive techniques (ART) help couples to have healthy children by mimicking physiological events. As intracytoplasmic sperm injection (ICSI) has been widely practiced among ARTs, sperm selection has become more relevant [6]. Sperm selection for intracytoplasmic sperm injection (ICSI) based on morphology and motility needs to be improved to increase ART’s success rate [7]. Since the genome and epigenome of spermatozoa are transmitted to the embryo, their impact on the embryo’s health is a topic of debate. Y chromosome microdeletions, aneuploidy, decreased protamination, and DNA methylome abnormalities can decrease embryo quality. Poor embryo quality decreases ART success rates and causes comorbidities among ART babies. Therefore, identifying molecular markers for sperm selection could increase ART success while reducing comorbidities [8].

Epigenetic modifications during testicular differentiation and spermatogenesis regulate sperm function and male fertility [9]. Primordial germ cells (PGC) are differentiated during testicular development. After puberty, spermatogenesis occurs, and spermatogonia starts a series of mitotic and meiotic divisions to give rise to spermatozoa. Sperm maturation in epididymis and capacitation in female genital tracts facilitate sperm function. All of these have an impact on the sperm epigenome [10, 11]. The alterations of sperm epigenome are related to the number, structure, and function of the sperm. Therefore, clinical research focuses on epigenetic alterations of the sperm and their relations with male infertility to develop personalized treatments [12, 13].

DNA methylation is a well-established epigenetic modification. DNA methyl transferases add a methyl group (-CH_3_) to the fifth carbon of cytosine. DNA methylation of the promoter inhibits gene expression [14, 15]. Genomic imprinting is the monoallelic gene expression from paternal or maternal chromosomes. Differentially methylated regions of the paternally imprinted gene are highly methylated on paternal chromosomes, and the maternal allele is used for gene expression [9, 16]. *H19* is a paternally imprinted gene transcribed to the long noncoding RNA (lncRNA). When the imprinting control region (ICR) of *H19* is methylated on the paternal chromosome, *H19* cannot be expressed. However, the same ICR methylation allows *IGF2* expression, a paternally expressed gene [17–19]. *H19/IGF2* methylation reduction is observed in the sperm of individuals with oligozoospermia and recurrent pregnancy loss. *IGF2* antisense (*IFG2AS*) is a lncRNA which is paternally expressed. Promotor methylation of *IGF2AS* at placental villi is associated with early pregnancy loss (EPL) [20]. A meta-analysis showed that sperm’s *H19* differently methylated region (DMR) methylation levels were significantly lower in infertile than fertile males. Conversely, *MEST* methylation was significantly higher in the infertile [21]. Despite the heterogeneity among the studies, *MEST* and *H19* have become the most prominent candidates for evaluating male infertility. During spermatogenesis, the methylation patterns of paternal and maternal imprinting genes change among spermatogenic cells. *H19* methylation appears in adult spermatogonia and is maintained in spermatozoa. *MEST* is a paternally expressed gene, is demethylated during fetal spermatogonia, and remains demethylated in spermatozoa [22]. *MESTIT1*, an intronic transcript of MEST, is an antisense RNA and also a paternally expressed gene. *MESTIT1* is an antisense RNA and a paternally expressed gene. Since *MESTIT1* is highly expressed in the testis and sperm, it is believed that it can have a regulatory function for fertility [23, 24]. Testis-specific gene A14 (*TSGA14)* and *MEST* are 7q autism susceptibility locus genes. Epigenetic modifications of the 7q locus are suggested to play a role in the etiopathogenesis of autism spectrum disorder [25]. *TSGA14* is not an imprinted gene [26] and is expressed into centrosomal protein 41 (CEP41). CEP41 is found in primary cilia, and its mutations are associated with the ciliopathy syndromes [27]. Although *CEP41* is highly expressed in human testis [26], the association with male infertility remains unknown. Another imprinted gene locus is *GNAS,* which contains multiple genes. *XLαs*, *A/B*, and *GNAS* antisense 1 (*GNASAS1)* are paternally expressed genes, but *NESP55* is maternally expressed from the *GNAS* locus. Although the impact of methylation of these genes on male infertility remains unclear, it was shown that ARTs affect the methylation of these genes in children [28–31].

Epigenetic changes in spermatozoa have an impact on fertilization and beyond. DNA methylation and transcripts of spermatozoa are part of epigenetic modifications [32, 33]. Methylation anomalies of the *H19*, *IGF2*, *GNAS*, *GNASAS*, and *MEST* imprinted genes have not previously been associated with male infertility based on transcriptome data. While *MESTIT1* and the non-imprinted gene *CEP41* are highly expressed in the testis, their effects on male fertility are still not fully understood. This study aims to evaluate how DNA methylation changes in *CEP41*, *MEST*, *MESTIT1*, *H19*, *IGF2AS*, and *GNASAS* of spermatozoa impact transcripts and male fertility.

## Materials and methods

### Study design

The study was conducted with the approval of the Gazi University Clinical Research Ethics Committee (25/02/2021, 04). Thirty males who had spontaneously impregnated spouses within 2 years were recruited to the fertile group. Sixty infertile males admitted to the Gazi University Hospital Assisted Reproductive Techniques Centre were divided into two groups based on sperm count: oligozoospermic (*n* = 30) and normal sperm count (*n* = 30). After assessing the total sperm count in infertile men, those with a total sperm count below 39 million were classified into the oligozoospermia group, while those with a count of 39 million or above were included in the normal sperm count group. Known male infertility etiologies, such as karyotype anomalies, hypogonadism, infections, testicular surgery, and irradiation, were exclusion criteria for both groups. After collecting demographic data, semen samples from the volunteers were used for semen analysis and further evaluations.

### Semen analysis and sperm isolation

Semen analysis was conducted according to the WHO guidelines [34]. Semen volume, sperm concentration, and motility were evaluated. Sperm morphology was assessed with Spermac stain [35]. Sperm concentrations were adjusted to less than 10 million/ml. The seminal plasma was discarded after centrifugation for 15 min at 2000 g. The pellet was then mixed with 1 ml PBS and centrifuged at 1600 g for 10 min. Subsequently, the pellet was incubated with SCLB (0.5% TritonX-100 in 0.1% SDS) for 30 min on ice. After centrifugation for 15 min at 1600 g, the supernatant was discarded. The pellet was rinsed with PBS before being separated into two for RNA and DNA extraction [36].

### Gene expression analysis

RNA extraction was conducted using TRIzol (Invitrogen, Thermo Fisher Scientific, MA, USA) according to the manufacturer’s protocol [37]. Sperm were mixed with 1 ml TRIzol and incubated at room temperature for 5 min. Subsequently, 200 μl chloroform was added and vortexed for 15 s. The mixture was allowed to rest for 2 min at room temperature and then centrifuged at 12, 000 g at + 4 °C for 15 min. The upper clear phase was transferred to the new tube. After adding 0.5 ml of isopropanol, the mixture was incubated overnight at − 20 °C. Centrifugation at 12,000 g was performed at + 4 °C for 10 min. After discarding the supernatant, 1 ml of 75% ethanol was added to the pellet and mixed by pipetting. The mixture was centrifuged at 7500 g at + 4 °C for 5 min. Following ethanol removal, the pellet was air-dried for 5 min. Finally, the RNA was dissolved in DEPC water. Qualification and quantification of RNA were conducted using the NanoDrop (Thermo Fisher Scientific, MA, USA).

To synthesize cDNA, the SensiFAST™ cDNA Synthesis Kit (Bioline, UK) was used. Up to 1 μg of RNA was mixed with 4 μl of TransAmp buffer and 1 μl of reverse transcriptase for each reaction. The mixture was then brought to a total volume of 20 μl with nuclease-free water. The synthesis occurred in three steps: 10 min at 25 °C for primer annealing, 15 min at 42 °C for reverse transcription, and 5 min at 85 °C for enzyme inactivation. Real-time PCR was performed using SensiFAST™ SYBR No-ROX Kit (Bioline, UK). For each reaction, 5 μl cDNA, 5 μl forward and reverse primers, and 10 μl of SensiFAST™ SYBR No-ROX mix were used. The primer sequences and melting temperatures are provided in Table 1. A two-step reaction protocol was applied. Following a 5-min polymerase activation at 95 °C, denaturation (95 °C, 10 s) and 30-s binding/elongation steps were repeated for 40 cycles at a separate temperature for each target gene (as indicated in Table 1). β-actin gene was used as the housekeeping gene. Gene expression analysis was performed among three groups using the Relative Expression Software Tool (REST©) [38].

**Table 1:** The primer sequences and melting temperatures for RT-PCR

Target gene	Forward sequence (5′–3′)	Reverse sequence (5′–3′)	Tm (°C)
MESTIT1	ACAACACAGGCCAGAAACAC	TCCAGTCTGCCCACTTGAT	65
MEST	TGTCAAATGGAGGTATCTTT	AAGTTCATCAGTCGTGTG	65
GNASAS	GAGATGAGTTTCTGAGGAT	CAGGTGAAATGAGGGTAG	62.5
CEP41	GATCATCCAAGTTGCTTCC	GCAGAATCATTGTCTTCCA	57
H19	CCTTCCTGAACACCTTAG	GTAGCACCATTTCTTTCAT	59
IGF2AS	GAAACAGCACTCCTCAAC	CAGACACCAATGGGAATC	61
ACTB	TGAAGATCAAGATCATTGCT	ATACTCCTGCTTGCTGAT	59

### DNA methylation analysis

Sperm DNA was extracted using the QuickGene DNA isolation kit (FUJIFILM, Japan). The spermatozoa suspension was mixed with 180 µl of tissue lysis buffer (MDT) solution. Later, 20 µl proteinase K was added. The mixture was vortexed for 15 s and incubated at 56 °C for 1 h. Then, 180 µl lysis buffer (LDT) was added and vortexed. The mixture was incubated at 70 °C for 10 min. Next, 240 µl 100% ethanol was added and mixed. The mixture was transferred to the tube of the Kurabo (FUJI) QuickGene Mini80 and was filtered. The filtered lysate was washed three times with 750 µl wash buffer (WDT). The extracted DNA was collected with 50 µl of elution buffer. DNA quality and quantity assessment were conducted using the NanoDrop (Thermo Fisher Scientific, MA, USA).

The EZ DNA Methylation-GoldTM kit (Zymo Research, USA) was used for DNA bisulfite modification [39]. Twenty microliters of DNA containing 200 to 300 ng was mixed with 130 µl CT converting reagent. The conversion took place at 98 °C for 10 min and 64 °C for 150 min. The mixture was then transferred after adding 600 µl M-binding buffer to the spin column and centrifuged at 10,000 g for 30 s. Subsequently, 100 µl M-washing buffer was added to the column and centrifuged at 10, 000 g for 30 s. After incubating with 200 µl M-desulphonation buffer for 15 min at room temperature, the mixture was centrifuged at 10,000 g for 30 s. The column was centrifuged at 10, 000 g for 30 s with M-wash buffer twice. DNA was collected using a 15 µl elution buffer (CTD).

The DNA methylation of targeted CpG regions was evaluated with pyrosequencing [39]. Bisulphite-modified DNA was amplified with PyroMark PCR kits (Qiagen, Germany). The reaction was initiated at 95 °C for 15 min and 45 cycles (substantially at 94 °C, 55 °C, and 72 °C for 30 s each). PyroMark CpG assays were utilized for PCR and pyrosequencing (Table 2). Once the amplicons were loaded onto the sepharose beads, sequence primers and beads were mixed in the annealing buffer. PyroMarkQ24 (Qiagen, Germany) was employed for pyrosequencing. The methylation percentage was determined using PyromarkQ24 Advanced Software (Qiagen, Germany).

**Table 2:** Pyromark CpG assay

Gene name	Gene globe ID	Sequence to analyze (5′–3′)	CpG #
MESTIT1	PM00685489	GACGGCTTTCGCAAAGCACCGGAGGCTCAGGGCCGCGTGTTTGGGGAAAACGGTTAGTCCTTTGAGCCGGC	7
MEST	PM00126742	AGCGCATGCGCAACCGGTTCTCCGA	4
GNASAS	PM00081039	CGGCGTCGCTGCAAGCCAAAGAAGCCCACCCGCCGT	5
CEP41	PM00126728	CCGTGAGGCTAGAACCCCGAACGTGGTCGGT	4
H19	PM00046830	TGGCSCGSCRGGGCGGTCTGGGCAGGGCCGCGT	4
IGF2AS	PM00046011	CGCGAGTTCGGTCTCGGGGGCCACCACGA	5

### Data analysis and statistics

DNA methylation cutoff values were obtained from ROC analysis for each gene to distinguish between infertility and fertility. The methylation status of each CpG site was categorized as either hypermethylated or hypomethylated based on these established cutoff values. Statistical tests were conducted using SPSS 28.0 (IBM, USA). ANOVA or Kruskal–Wallis tests were employed to determine differences between groups based on data distribution, with Bonferroni and Tamhane post-hoc tests applied. Correlations between parameters were assessed using Spearman’s test. Multinomial logistic regression was performed to determine the association between infertility and hypermethylation of the genes. A significance level of *p* < 0.05 was considered statistically significant.

## Results

The demographic characteristics of the fertile, oligozoospermic, and normal sperm count groups are represented in Table 3. The differences in age and body mass index (BMI) between the groups were statistically insignificant. Additionally, there was no significant difference between the groups in terms of sexual abstinence and semen volumes. The age-matched groups showed significant differences in semen analysis (Table 3). The oligozoospermic group had significantly lower total sperm count and sperm concentration than the fertile and normal sperm count groups. All groups were within the normal limits regarding motility. The normal sperm count group had significantly higher motility than the fertile and oligozoospermic groups. The fertile group had significantly higher normal morphology than the oligozoospermic and normal sperm count groups.

**Table 3:** Comparisons of the fertile, the oligozoospermic, and the normal sperm count groups regarding demographic characteristics and semen analysis

	Fertile	Oligozoospermic	Normal sperm count	*p* values	*p*1	*p*2	*p*3
Age^a^, year	37.63 ± 4.72	35.26 ± 7.26	34.43 ± 6.21	0.119			
Body Mass Index^a^, kg/m^2^	25.81 ± 1.68	26.03 ± 2.07	25.70 ± 2.17	0.803			
Sexual abstinence^b^, days	3 (1)	3 (1)	3 (2)	0.295			
Semen volume^b^, ml	3 (1)	3 (1.38)	3 (2)	0.118			
Sperm concentration^b^, million/ml	35.5 (33.75)	10.5 (10)	50 (15)	**0.0001**	**0.0001**	**0.0001**	0.232
Total sperm count^b^, million	101.5 (112.38)	27.5 (35.38)	130.25 (87.63)	**0.0001**	**0.0001**	**0.0001**	0.150
Sperm motility^b^, %	55 (9.75)	56 (10)	64(15)	**0.009**	0.367	**0.037**	**0.003**
Normal morphology^b^, %	5 (2)	0 (0.75)	0 (2)	**0.0001**	**0.0001**	0.232	**0.0001**

^a^Normally distributed data is presented as mean ± standard deviation, and ANOVA was used for statistical analysis

^b^Non-normally distributed data is presented as median (interquartile range), and the Kruskal–Wallis test was used for statistical analysis

*P* values were determined using Tamhane’s test and are represented as *p*. Specifically, *p* compares all groups, *p1* compares fertile vs oligozoospermic, *p*2 compares oligozoospermic vs normal sperm count, and *p*3 compares fertile vs normal sperm count. *P* values < 0.05 are bolded

**Fig. 1:**
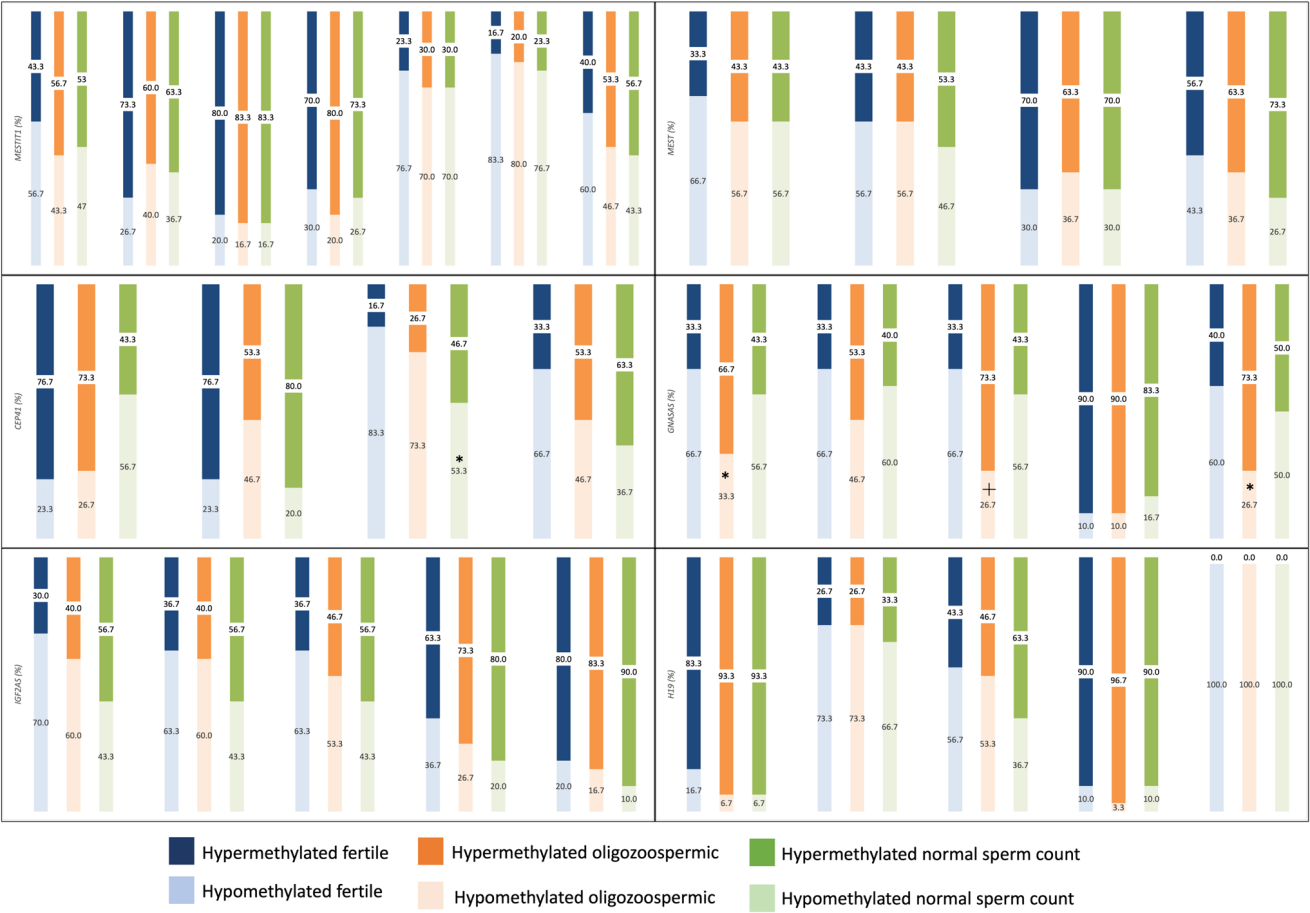
Frequencies of hypermethylation (dark color) and hypomethylation (light color) for each CpG dinucleotides. Blue bars represent the fertile group, orange bars represent the oligozoospermic group, and green bars represent the normal sperm count group. The statistically significant changes are compared with the fertile group indicated with “*p < 0.05” and “†*p* < 0.01.”

The methylation cutoff values for each gene were determined using ROC analysis based on fertile or infertile subjects (Table 4). The area under the ROC curve was statistically significant for each gene. Methylation levels below 87.5% of each CpG site of the paternally imprinted *H19* were categorized as hypomethylated. CpGs of *MEST* (4.5%), *MESTIT1* (14.5%), *GNASAS* (6.5%), *CEP41* (3.5%), and *IGF2AS* (5.5%) were categorized as hypermethylated if methylation levels were above their respective cutoff values. The frequency of hypomethylated *H19* and hypermethylated genes was compared among the fertile, oligozoospermic, and normal sperm count groups (Fig. 1). *GNASAS* was significantly hypermethylated at the 1st (66.7%), 3rd (73.3%), and 5th (73.3%) CpG sites in the oligozoospermic group compared to the fertile group (1st 33.3%; 3rd 33.3%; and 5th 40%). *CEP41* was significantly hypermethylated at the 3rd CpG site in the normal sperm count group (46.7%) compared to the fertile group (16.7%). Methylation changes at *MEST*, *MESTIT1*, *H19*, and *IGF2AS* CpG sites among groups were statistically insignificant. Multinomial logistic regression analysis was performed to predict infertility with hypermethylation of *GNASAS* and *CEP41* (Table 5). Infertility with normal sperm count was predicted by *CEP41* hypermethylation (OR = 1.750, 95% CI 1.038–2.950) and hypermethylation of both *CEP41* and *GNASAS* (OR = 2.389, 95% CI 1.137–5.021). Oligozoospermic infertility was predicted solely by *GNASAS* hypermethylation (OR = 2.460, 95% CI 1.315–4.603).

**Table 4:** The cutoff methylation level, sensitivity, specificity, AUC, 95% CI, and *P* values of each gene to distinguish fertility and infertility were determined by the ROC analysis

Gene	Cutoff methylation level	Sensitivity	Specificity	AUC	95% CI	*P* values
MESTIT1	0.145	0.56	0.533	0.554	0.506–0.601	**0.027**
MEST	0.045	0.567	0.492	0.567	0.504–0.630	**0.039**
GNASAS	0.065	0.617	0.54	0.589	0.535–0.644	**0.002**
CEP41	0.035	0.642	0.483	0.597	0.535–0.659	**0.003**
H19	0.875	0.543	0.513	0.558	0.501–0.615	**0.044**
IGF2AS	0.055	0.623	0.507	0.580	0.523–0.638	**0.005**

*AUC*, area under curve; *CI*, confidence interval

*P* values < 0.05 are bolded

**Table 5:** Multinominal logistic regression analysis to predict infertility

Group^a^		B	SE	Wald	*p*-value	OR	95% confidence interval Lower bound Upper bound	
Oligozoospermic	Intercept	−0.588	0.211	7.774	0.005			
	GNASAS hypermethylation	0.900	0.320	7.931	**0.005**	2.460	1.315	4.603
	CEP41 hypermethylation	0.237	0.261	0.829	0.363	1.268	0.761	2.113
	GNASAS hypermethylation plus CEP41 hypermethylation	0.176	0.343	0.264	0.608	1.192	0.609	2.334
Normal sperm count	Intercept	−0.100	0.183	0.300	0.584			
	GNASAS hypermethylation	−0.305	0.342	0.799	0.371	0.737	0.377	1.440
	CEP41 hypermethylation	0.560	0.266	4.412	**0.036**	1.750	1.038	2.950
	GNASAS hypermethylation plus CEP41 hypermethylation	0.871	0.379	5.280	**0.022**	2.389	1.137	5.021

^a^The reference category is fertile. *P* values < 0.05 are bolded

Abbreviations: *B*, the logistic coefficient; *SE*, standard error; *OR*, odds ratioss

**Fig. 2:**
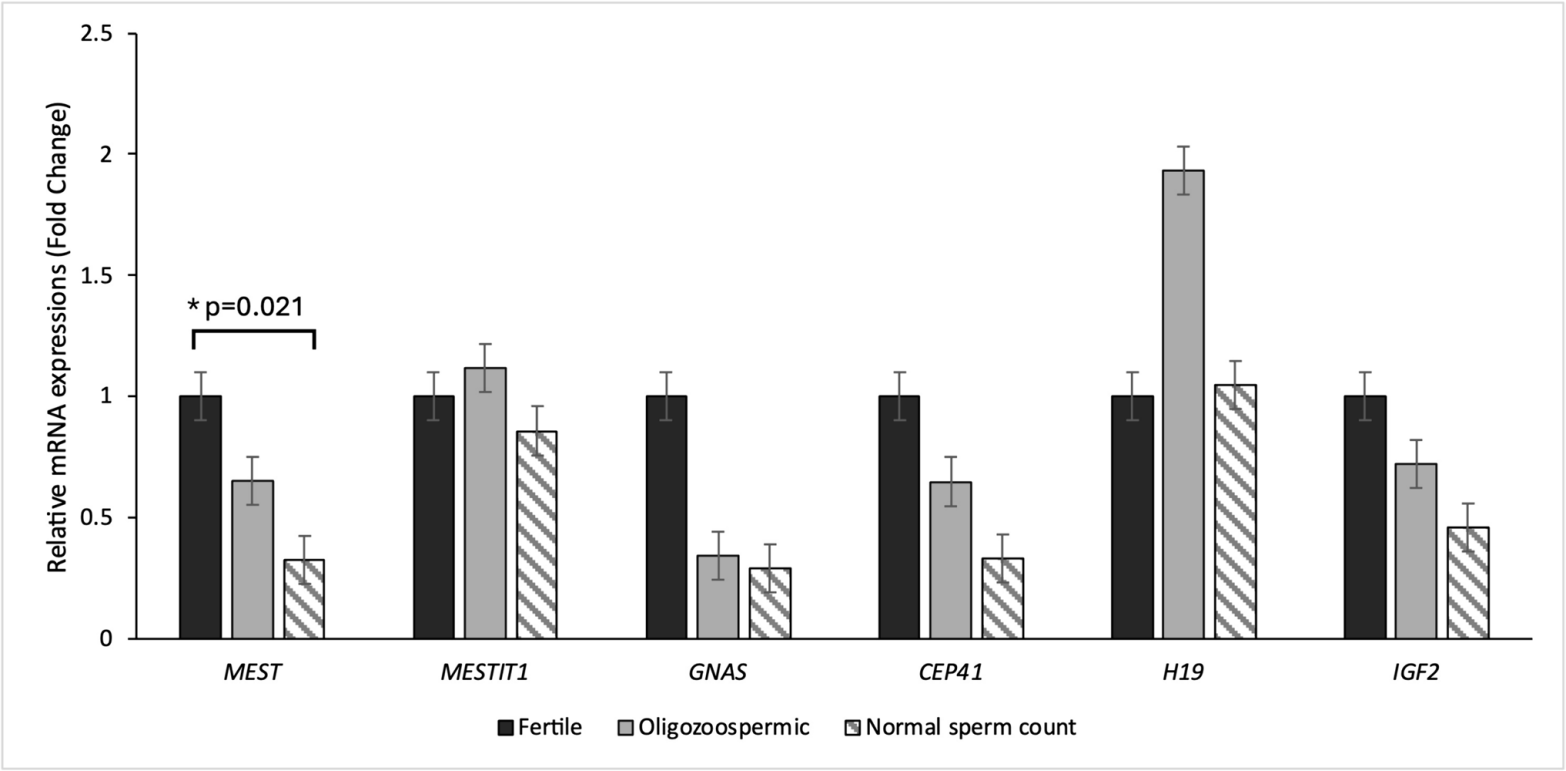
Comparison of *CEP41*, *MEST*, *MESTIT1*, *H19*, *IGF2AS*, and *GNASAS* gene expression fold changes between oligozoospermic and normal sperm count individuals with the fertile group. *p = 0.021

Comparison *CEP41*, *MEST*, *MESTIT1*, *H19*, *IGF2AS*, and *GNASAS* gene expressions are demonstrated in Fig. 2. The normal sperm count group exhibited significantly lower expression (approximately 3.4-fold change) of *MEST* compared to the fertile group. While *MESTIT1*, *GNASAS*, *CEP41*, and *IGF2AS* transcripts were lower in the normal sperm count group compared to the fertile group, these differences were statistically insignificant. *H19* expression was higher in the normal sperm count group than in the fertile group. The oligozoospermic group showed statistically insignificant increases in *H19* and *MESTIT1* expressions compared to the fertile group. *MEST*, *GNASAS*, *CEP41*, and *IGF2AS* mRNA expression levels were lower in the oligozoospermic group than in the fertile group. However, these decreased expressions were not found to be statistically significant. Although the gene expressions of all genes were decreased in the normal sperm count group compared to the oligozoospermic group, it was not statistically significant.

The correlations between parameters were evaluated among the fertile, oligozoospermic, and normal sperm count groups (Supplementary Table 1). mRNA expression of *MEST* was significantly negatively correlated with the 1st (*r* =  − 0.517) and 2nd (*r* =  − 0.510) CpGs of *MEST* in the normal sperm count groups. Conversely, these associations were absent in the fertile and oligozoospermic groups. In the fertile group, mRNA expression of *IGF2* was significantly negatively correlated with *H19* methylation at the 1st (*r* =  − 0.367), 2nd (*r* =  − 0.379), and 4th (*r* =  − 0.469) CpGs. These significant correlations were not observed among the infertile groups. Additionally, *IGF2* mRNA expression in the fertile group was significantly correlated with *IGF2AS* methylation at the 2nd CpG (*r* = 0.388), while this correlation was not present in the infertile groups. In the fertile group, there was no significant correlation found between the mRNA expressions of *GNAS*, *MESTIT1*, and *CEP41* and the methylation levels of these genes. mRNA expressions of *MESTIT1*, *MEST*, and *CEP41* were significantly positively correlated among all groups. *IGF2* and *H19* mRNA expressions were also significantly correlated among all groups. mRNA expression of *GNASAS* was significantly correlated with *H19* in the oligozoospermic group (*r* = 0.641), but not in the normal sperm count or fertile groups (Table 6).

**Table 6:** Correlation coefficients (*r*) of different parameters among the fertile, the oligozoospermic, and the normal sperm count groups

1st parameter	2nd parameter	Fertile	Oligozoospermic	Normal sperm count
*MEST* expression	MEST_ CpG1 methylation	*r* = 0.180	*r* = − 0.280	*r* = − 0.517^**^
*MEST* expression	MEST _ CpG2 methylation	*r* = − 0.019	*r* = − 0.175	*r* = − 0.510^**^
*MEST* expression	MEST _ CpG3 methylation	*r* = 0.206	*r* = − 0.252	*r* = − 0.080
*MEST* expression	MEST _ CpG4 methylation	*r* = 0.089	*r* = − 0.252	*r* = − 0.092
*IGF2* expression	IGF2AS_ CpG1 methylation	*r* = 0.088	*r* = 0.236	*r* = − 0.249
*IGF2* expression	IGF2AS_ CpG2 methylation	*r* = 0.388^*^	*r* = 0.189	*r* = − 0.342
*IGF2* expression	IGF2AS_ CpG3 methylation	*r* = 0.252	*r* = 0.324	*r* = − 0.187
*IGF2* expression	IGF2AS_ CpG4 methylation	*r* = 0.196	*r* = 0.131	*r* = − 0.010
*IGF2* expression	IGF2AS_ CpG5 methylation	*r* = 0.091	*r* = 0.264	*r* = 0.032
*IGF2* expression	H19_ CpG1 methylation	*r* = − 0.367^*^	*r* = − 0.185	*r* = − 0.193
*IGF2* expression	H19_ CpG2 methylation	*r* = − 0.379^*^	*r* = 0.152	*r* = − 0.290
*IGF2* expression	H19_ CpG3 methylation	*r* = − 0.326	*r* = − 0.050	*r* = − 0.128
*IGF2* expression	H19_ CpG4 methylation	*r* = − 0.469^**^	*r* = − 0.011	*r* = − 0.116
*H19* expression	H19_ CpG1 methylation	*r* = − 0.269	*r* = − 0.054	*r* = − 0.170
*H19* expression	H19_ CpG2 methylation	*r* = − 0.257	*r* = 0.261	*r* = − 0.016
*H19* expression	H19_ CpG3 methylation	*r* = − 0.210	*r* = 0.073	*r* = − 0.132
*H19* expression	H19_ CpG4 methylation	*r* = − 0.283	*r* = 0.032	*r* = − 0.071
*GNAS* expression	GNASAS_ CpG1 methylation	*r* = 0.155	*r* = 0.291	*r* = 0.241
*GNAS* expression	GNASAS_ CpG2 methylation	*r* = 0.155	*r* = 0.325	*r* = 0.130
*GNAS* expression	GNASAS_ CpG3 methylation	*r* = 0.155	*r* = 0.109	*r* = − 0.058
*GNAS* expression	GNASAS_ CpG4 methylation	*r* = 0.032	*r* = − 0.116	*r* = 0.000
*GNAS* expression	GNASAS_ CpG5 methylation	*r* = − 0.071	*r* = 0.218	*r* = − 0.166
*CEP41* expression	CEP41_ CpG1 methylation	*r* = 0.214	*r* = − 0.013	*r* = 0.351
*CEP41* expression	CEP41_ CpG2 methylation	*r* = 0.170	*r* = − 0.155	*r* = 0.144
*CEP41* expression	CEP41_ CpG3 methylation	*r* = − 0.165	*r* = 0.000	*r* = 0.259
*CEP41* expression	CEP41_ CpG4 methylation	*r* = − 0.237	*r* = 0.263	*r* = 0.024
*IGF2* expression	*H19* expression	*r* = 0.613^**^	*r* = 0.808^**^	*r* = 0.534^**^
*MESTIT1* expression	*MEST* expression	*r* = 0.553^**^	*r* = 0.839^**^	*r* = 0.705^**^
*MESTIT1* expression	*CEP41* expression	*r* = 0.577^**^	*r* = 0.670^**^	*r* = 0.409^*^
*CEP41* expression	*MEST* expression	*r* = 0.888^**^	*r* = 0.789^**^	*r* = 0.495^**^
*GNAS* expression	*MEST* expression	*r* = 0.888^**^	*r* = 0.841^**^	*r* = 0.445^**^
*GNAS* expression	*H19* expression	*r* = 0.212	*r* = 0.641^**^	*r* = 0.190

The statistically significant correlations are signed with ^*^p < 0.05 and ^**^p < 0.01

## Discussion

The relationship between male infertility and the DNA methylation of spermatozoa is the topic of various research [40]. In the face of the diverse factors influencing the DNA methylome of the spermatozoa, there are not any findings which have yet been confirmed by multiple studies [41]. El Hajj et al. [42] compared spermatozoa from infertile males with controls and found that the methylation levels of *NESPAS*, also known as *GNASAS*, which is a member of the *GNAS* locus, as well as *MEST* and *H19*, did not show statistically significant differences. In our study, the methylation levels of *MEST* and *H19* did not change significantly among the three groups, consistent with the findings of El Hajj et al. [42]. On the other hand, we found significant hypermethylation of three CpGs in *GNASAS* in the oligozoospermic group compared to the fertile group. Our different findings may be related to inclusion criteria; for example, we included fertile males as controls, whereas the control group in El Hajj et al. [42] consisted of presumably fertile males who underwent infertility treatment due to female factor infertility and repeatedly exhibited normal semen parameters without a cause for male infertility. On the other hand, we observed that El Hajj et al. [42] used a previous edition of the guidelines compared to ours, leading to the application of different reference values [42]. Moreover, we determined the hypermethylation rates based on a cutoff value, whereas El Hajj et al. [42] used the methylation percentage directly. To determine the cutoff value with the ROC analysis, we choose higher sensitivity and lower specificity to more accurately evaluate the infertile individuals [43]. Based on the cutoff value we utilized, we found that *GNASAS* hypermethylation could serve as a significant predictor of oligozoospermic infertility. Even though we demonstrated no significant difference in *MEST* methylation among groups, similar to El Hajj et al. [42], the significant downregulation of *MEST* in the normal sperm count group in our study was a novel finding not reported in the literature. Decreased levels of *MEST* mRNA might result from methylation abnormalities of different CpGs in the *MEST* promoter region or chromatin remodelling errors that inhibit transcription factor binding in the spermatogonia subpopulation of our normal sperm count group. Post-transcriptional mRNA degradation is another possible explanation for our findings. It is our understanding that spermatozoa of men with normal sperm count may fail in fertilization or embryo development due to differences in mRNA expression levels, specifically in their downregulation, which do not affect sperm count.

Becker et al. [44] found lower *CEP41* expression of the spermatozoa in oligoasthenozoospermia than in the controls. Unlike Becker et al. [44], we observed no significant downregulation of *CEP41* expression in both the oligozoospermic and normal sperm count groups compared to the control group. The discrepancy between our results and Becker’s findings may stem from variations in inclusion criteria. Their experiment group included solely oligoastenozoospermic males while we had normal sperm count and oligozoospermic group separately. Additionally, while Becker et al. [44] demonstrated miRNA-mediated downregulation of gene expression in spermatozoa, our study focused on investigating *CEP41* methylation as a distinct epigenetic control mechanism. In our study, although the downregulation of *CEP41* expression in the normal sperm count group was not statistically significant, *CEP41* was significantly hypermethylated in the normal sperm count group compared to the controls (*p* < 0.05). We believe that the decrease in *GNASAS* and *CEP41* transcription in both normal sperm count and oligozoospermia groups may be attributed to the statistically significant hypermethylation percentage of these genes that we identified. Additionally, various epigenetic factors during spermatogenesis, such as histone modifications, chromatin restructuring, or methylation changes at CpGs different from those we detected, may also have contributed to the decrease in transcription levels. We demonstrated that *CEP41* hypermethylation alone and in combination with *GNASAS* hypermethylation is a marker for assessing infertility with normal sperm count. On the other hand, for assessing oligozoospermic infertility, *CEP41* hypermethylation did not increase the predictive value of *GNASAS* hypermethylation. *GNASAS* hypermethylation might affect spermatozoa number, whereas *CEP41* hypermethylation might instead affect sperm function.

Oligozoospermic patients have lower live birth rates, and it is believed that sperm methylome anomalies have an impact on the success of assisted reproductive technology (ART) [45]. Although we demonstrated that oligozoospermic males had *GNASAS* hypermethylation, its impact on embryos was beyond the scope of our research. Several studies have shown that babies born through ART may exhibit DNA methylation abnormalities, but not all findings were statistically significant [46, 47]. Katari et al. [48] showed that *GNAS* methylation changes due to ART were detected in cord blood but not in the placenta. Melamed et al. [49] also showed that *GNAS* in cord blood of ART babies exhibited three differentially methylated CpG sites. Among naturally conceived babies, Tobi et al. [45] showed that *GNASAS* methylation levels did not change significantly between babies who were small for gestational age (SGA) and the appropriate for gestation age. Given the frequent use of ART for oligozoospermic patients, further research is needed to elucidate the effects of *GNASAS* hypermethylation, which we detected in the oligozoospermic group, on pregnancy outcomes.

Unlike significant hypermethylation of *CEP41* and *GNASAS* in infertile males, we did not find statistically significant methylation differences of *MEST*, *MESTIT1*, *IGF2AS*, and *H19*. Marques et al. [50] detected aberrant methylation of *H19* and *MEST* in sperm from oligozoospermic patients; however, they did not include a fertile group for comparison. They showed abnormalities in *H19* and *MEST* methylation in testicular spermatozoa, but again, the study did not include a fertile control group [51]. Unlike our findings, which are derived from four CpGs of *MEST*, Hammond et al. [52] reported significant hypermethylation of *MEST* on one of eighteen CpG sites in oligozoospermic males compared to fertile males. Amjadian et al. [53] found higher *MEST* methylation in oligoasthenoteratospermia than in asthenospermia and normospermia; however, their groups were constructed differently from ours. On the other hand, in contrast to Amjadian et al. [53], Song et al. [54] demonstrated significant *MEST* hypomethylation in the asthenozoospermic group compared to normozoospermic infertile males. Montjean et al. [55] identified methylation abnormalities in *MEST* and *H19* among infertile males, but unlike us, they did not enroll fertile males for comparison. The differences in our findings could be attributed to various factors such as sample size, group composition, and the specific CpGs we evaluated. Recent meta-analyses focusing on *MEST* [56] and *H19* [9] indicated that methylation studies in male infertility reveal diverse results due to the heterogeneity among the studies.

In our study, *MEST* expression was significantly downregulated in the normal sperm count male; even the *MEST* methylation did not change significantly between groups. While MEST expression was significantly downregulated in individuals with MEST hypermethylation in the normal sperm count group, this correlation was not found in the fertile and oligozoospermic groups. Leitao et al. [57] investigated MEST methylation changes over 200 CpGs and showed altered methylation ranges in oligozoospermic men. Methylation of other CpGs in *MEST*, which we did not investigate, could be effective for *MEST* expression in our fertile group. Decreased *MEST* mRNA levels might result from increased mRNA degradation, as well as from inhibition of gene expression by non-coding RNAs. The mRNAs of spermatozoa had a testicular origin, and some of them were degraded by miRNAs throughout sperm’s transit within the male and female genital tract [32]. In addition, spermatozoa may become transcriptionally inactive after chromatin condensation [33]. Laurentino et al. [58] suggested that defects in MEST demethylation in primordial germ cells (PGCs) after migration during embryological development or aberrations in remethylation during spermatogenesis might lead to abnormal spermatozoa subpopulations. Marques et al. [59] showed that *MEST* demethylation continues in all spermatogenic cells from spermatogonia to spermatids. Another hypothesis to explain the findings in the normal sperm count group of our study could be the presence of an abnormally methylated spermatogenic subpopulation.

There have been studies investigating the impact of gene methylation and expression imprinting on pregnancy outcomes. Placental *MEST* hypermethylation and downregulation of gene expression were revealed among the early onset preeclampsia patients [60]. Similar to that*, IGF2AS* methylation was increased, and expression was downregulated in embryonic villi samples from early pregnancy loss [20]. Alterations in *IGF2AS* methylation were found in groups with deficient birth weight, persisting even after years [61]. In our study, *IGF2AS* methylation was not significantly different among the fertile, oligozoospermic, and normal sperm count groups. However, *IGF2* expression was significantly upregulated in fertile individuals whose 2nd CpG of *IGF2AS* was hypermethylated, unlike in the infertile group. Darbandi et al. [62] stated that *H19* differentially methylated region (DMR) methylation of the paternal chromosome has a reciprocal effect as downregulated *H19* expression and upregulated *IGF2* expression. In contrast to Darbandi et al. [62], we found a significant negative correlation between *IGF2* expression and *H19* methylation in the fertile group. These relations were lost among infertile groups. The positive correlation between *H19* and *IGF2* transcripts in our three groups suggested that spermatozoa might be associated with other epigenetic modifications rather than DNA methylation. Since *H19* methylated during spermatogenesis to transfer paternal imprinting for offsprings, *H19* and *IGF2* transcription should be completed ahead of DNA methylation [32]. The absence of significant correlations within the infertile groups in our study might be related to other disrupted epigenetic mechanisms, such as changes in ncRNAs, which are also important for embryo development and transgenerational inheritance.

In conclusion, the DNA methylome of spermatozoa significantly impacts male infertility. Despite limitations such as the scarcity of CpG sites in methylation analysis, our study is the first to demonstrate hypermethylation of *GNASAS* and *CEP41* in spermatozoa and to demonstrate the variable relationship between DNA methylation and gene expression in infertile men. *GNASAS* and *CEP41* hypermethylation should be evaluated in the further studies in order to develop more effective molecular markers in the diagnosis of male infertility, to elucidate the epigenetic regulation during spermatogenesis, and to reveal the effects of paternal epigenome on ART’s success and the health of new generations.

## Supplementary Information

Below is the link to the electronic supplementary material.

Supplementary file1 (XLSX 185 KB)

## Data Availability

All of the data of our study are available in Tables 1, 2, 3, 4, 5 and 6, Figures 1 and 2 and our supplementary file, and can be used by reference after our publication is published in the literature.
